# K_0.78_Na_0.22_MoO_2_AsO_4_

**DOI:** 10.1107/S1600536813018540

**Published:** 2013-07-10

**Authors:** Raja Jouini, Chahira Bouzidi, Mohamed Faouzi Zid, Ahmed Driss

**Affiliations:** a Laboratoire de Matériaux et Cristallochimie, Faculté des Sciences de Tunis, Université de Tunis El Manar, 2092 ElManar II Tunis, Tunisia

## Abstract

The title compound, potassium sodium dioxidomolybden­um(VI) arsenate, K_0.78_Na_0.22_MoO_2_AsO_4_, was synthesized by a solid-state reaction route. The structure is built up from corner-sharing MoO_6_ octa­hedra and AsO_4_ tetra­hedra, creating infinite [MoAsO_8_]_∞_ chains running along the *b*-axis direction. As, Mo and all but one O atom are on special positions (4*c*) with *m* symmetry and K (occupancy 0.78) is on a position (4*a*) of -1 in the tunnels. The possible motion of the alkali cations has been investigated by means of the bond-valance sum (BVS) model. The simulation shows that the Na^+^ motion appears to be easier mainly along the *b*-axis direction. Structural relationships between the different compounds of the *A*MoO_2_AsO_4_ (*A* = Ag, Li, Na, K, Rb) series and *MX*O_8_ (*M* = V; *X* = P, As) chains are discussed.

## Related literature

For background to the physico-chemical properties of related compounds, see: Gueho *et al.* (1993 ▶); Piffard *et al.* (1985 ▶). For details of structurally related compounds, see: Zid & Jouini (1996 ▶, 1999 ▶); Ben Hlila *et al.* (2009 ▶); Hajji & Zid (2006 ▶); Linnros (1970 ▶); Zid *et al.* (1997 ▶); Hajji *et al.* (2004 ▶); Belkhiri *et al.* (2012 ▶); Benhamada *et al.* (1991 ▶); Boudin *et al.* (1995 ▶). For the preparation, see: Jouini *et al.* (2012 ▶, 2013 ▶). For BVS pathway simulation, see: Ouerfelli *et al.* (2007 ▶); Ben Smida *et al.* (2013 ▶); Ben Amor *et al.* (2008 ▶). For bond-valence-sum calculations, see: Brown & Altermatt (1985 ▶).

## Experimental

### 

#### Crystal data

K_0.78_Na_0.22_MoO_2_AsO_4_
*M* *_r_* = 302.47
Orthorhombic, 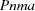

*a* = 10.5672 (9) Å
*b* = 6.6323 (8) Å
*c* = 6.9621 (8) Å
*V* = 487.94 (9) Å^3^
*Z* = 4
Mo *K*α radiation
μ = 10.05 mm^−1^
*T* = 298 K
0.45 × 0.33 × 0.23 mm

#### Data collection

Enraf–Nonius CAD-4 diffractometer
Absorption correction: ψ scan (North *et al.*, 1968 ▶) *T*_min_ = 0.025, *T*_max_ = 0.098
1413 measured reflections
576 independent reflections
553 reflections with *I* > 2σ(*I*)
*R*_int_ = 0.037
2 standard reflections every 120 min intensity decay: 1.2%

#### Refinement

*R*[*F* ^2^ > 2σ(*F* ^2^)] = 0.025
*wR*(*F* ^2^) = 0.067
*S* = 1.17
576 reflections
64 parameters
1 restraint
Δρ_max_ = 0.64 e Å^−3^
Δρ_min_ = −1.13 e Å^−3^

### 

Data collection: *CAD-4 EXPRESS* (Duisenberg, 1992 ▶; Macíček & Yordanov, 1992 ▶); cell refinement: *CAD-4 EXPRESS*; data reduction: *XCAD4* (Harms & Wocadlo, 1995 ▶); program(s) used to solve structure: *SHELXS97* (Sheldrick, 2008 ▶); program(s) used to refine structure: *SHELXL97* (Sheldrick, 2008 ▶); molecular graphics: Diamond (Brandenburg, 1998 ▶); software used to prepare material for publication: *WinGX* publication routines (Farrugia, 2012 ▶).

## Supplementary Material

Crystal structure: contains datablock(s) I, global. DOI: 10.1107/S1600536813018540/br2228sup1.cif

Structure factors: contains datablock(s) I. DOI: 10.1107/S1600536813018540/br2228Isup2.hkl

Additional supplementary materials:  crystallographic information; 3D view; checkCIF report

## Figures and Tables

**Fig. 1.**
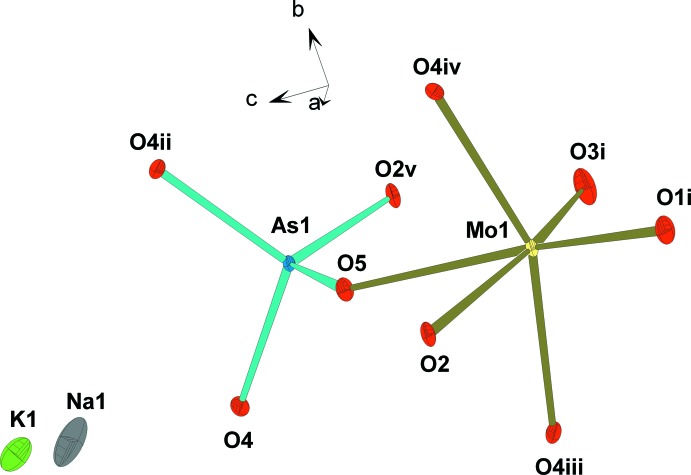
: Unité asymétrique dans K0.78Na0.22MoO2AsO4 Les ellipsoïdes ont été définis avec 50% de probabilités. [codes de symétrie:(i) x, y, -1 + z; (ii) x, 1/2 - y, z; (iii) 1/2 - x, -y, -1/2 + z; (iv) 1/2 - x, 1/2 + y, -1/2 + z; (v) -1/2 + x, 1/2 - y, 1/2 - z].

**Fig. 2.**
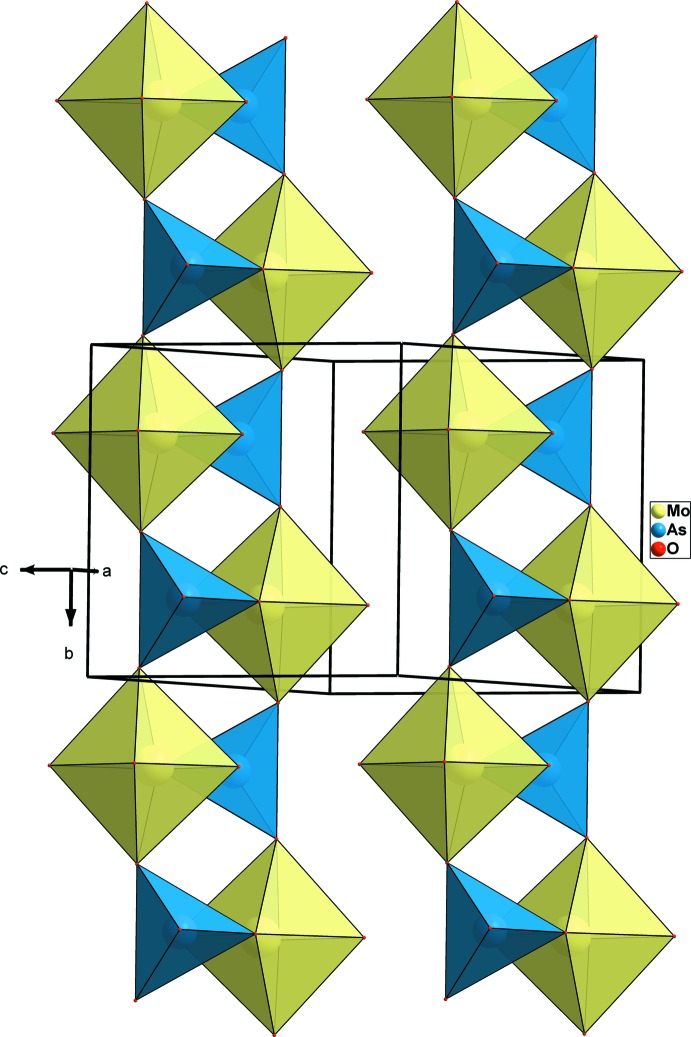
: Projection des chaînes doubles Mo2As2O14 selon la direction [010].

**Fig. 3.**
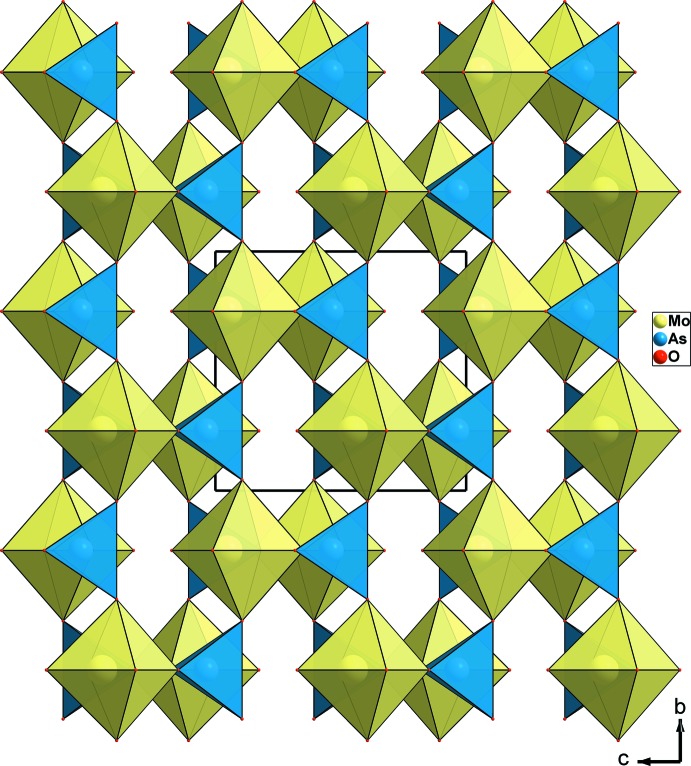
: Projection des rubans disposés parallèlement à la direction b.

**Fig. 4.**
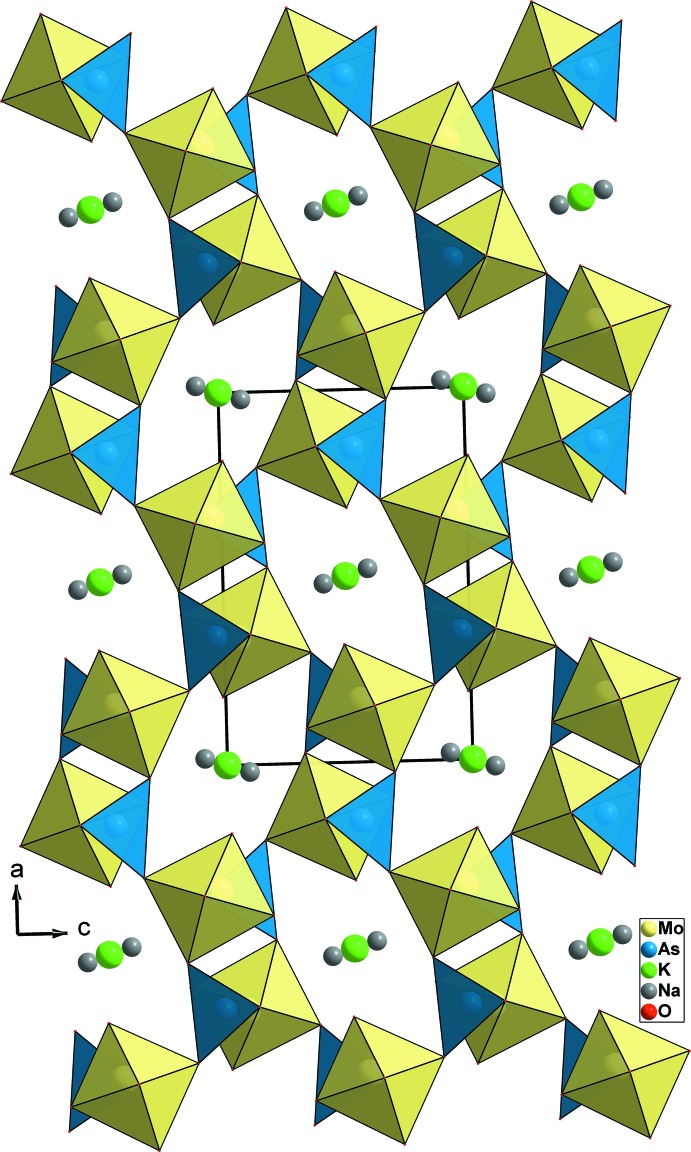
: Projection de la structure de K0.78Na0.22MoO2AsO4 selon b, mettant en évidence les canaux où logent les cations K+ et Na+.

**Fig. 5.**
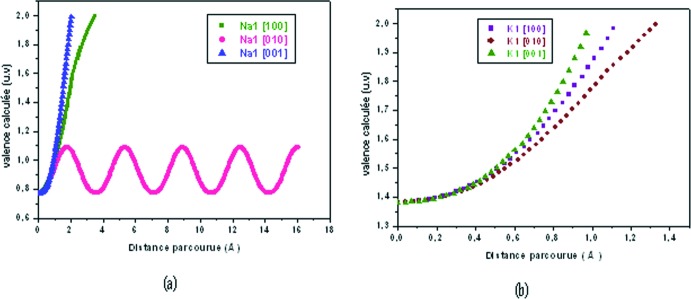
: Variation de la valence en fonction de la distance parcourue pour les cations: (a) Na+, (b) K+.

**Fig. 6.**
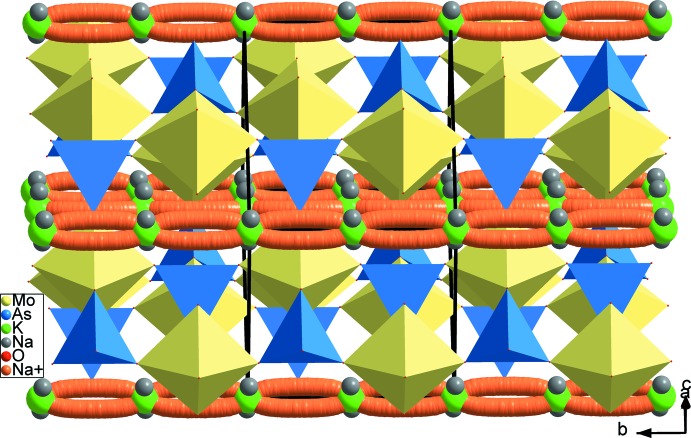
: Modélisation du chemin de conduction des cations Na+ selon b.

## supplementary crystallographic information

### Comment

Plusieurs travaux ont été consacrés à l'étude des matériaux inorganiques à charpentes ouvertes formées de tétraèdres et d'octaèdres. En effet, ces composés constituent un champ prometteur pour diverses applications. Ils possèdent aussi des propriétés de conduction ionique (Gueho *et al.*, 1993) et d'échange d'ions (Piffard *et al.*, 1985) assez performantes.

A titre de contribution à l'étude de ces types de matériaux, nous avons poursuivi l'exploration des systèmes A—Mo—As—O (A= Na, K) dans lesquels nous avons pu précédemment isoler les phases suivantes: Na_2_MoO_2_As_2_O_7_ (Jouini *et al.*, 2012) et K(MoO_2_)_4_O_3_(AsO_4_) (Jouini *et al.*, 2013).

Nos tentatives de synthèse, nous ont permis de trouver un matériau mixte de potassium et de sodium de formulaion K_0.78_Na_0.22_MoO_2_AsO_4_. Ce dernier appartenant à la famille des monoarséniates de formulation AMoO_2_AsO_4_ (A= cation monovalent).

L'unité *asym*étrique dans la structure est construite à partir d'un octaèdre MoO_6_ et d'un tétraèdre AsO_4_ partageant un sommet (Fig. 1). Ces unités se lient les unes aux autres selon la direction [010] formant des chaînes de formulation (MoAsO_8_)_∞_. Ces dernières s'associent par paires pour former des doubles chaînes de type Mo_2_As_2_O_14_ disposées selon la direction b (Fig. 2). Une disposition particulière de ces doubles chaînes permet, par mise en commun de sommets entre polyèdres de nature différente, de développer des rubans parallèles à la direction b (Fig. 3). La jonction de ces derniers par formation de ponts mixtes Mo—O—As conduit à une charpente tridimensionnelle possédant de larges canaux où résident les cations monovalents (Fig. 4).

Le calcul des différentes valences des liaisons utilisant la formule empirique de Brown (Brown & Altermatt, 1985) conduit aux valeurs suivantes: Mo1(6,109), As1(5,048) et Na1(0,935) ce qui confirme les degrés d'oxydation des différents ions dans la phase étudiée. Le concept BVS permet de déterminer les chemins de migration les plus probables des cations alcalins. Ce concept est détaillé dans des travaux antérieurs (Ouerfelli *et al.*, 2007; Ben Smida *et al.*, 2013). Ce modèle permet de montrer que la direction [010] est la plus favorable pour la mobilité du sodium. En effet, la valence maximale V_max_ selon la direction b est au voisinge de 1,05 u.v. (unité de valence) pour une distance de migration de l'ordre de 16 Å (Fig. 5a). Pour les autres directions, les valences calculées sont élevées (V_max_ > 2 u.v.) pour une distance courte de migration de 2 Å, le cation mobile rencontre donc des barrières énergétiques excercées par la charpente anionique. Par contre, le potassium de grande taille, occupant une position *sp*éciale est piégé dans les canaux. En effet, pour des distances courtes parcourues de l'ordre de 1 Å, la valence calculée est de l'ordre de 2 u.v. (Fig. 5 b). Ainsi pour migrer d'une position à une autre plus proche selon cette direction [010], le cation K^+^ doit franchir un mur de potentiel de valence maximale de 2 u.v. pour cette direction de migration. Par conséquent, il peut diminuer aussi la mobilité des cations Na^+^. Notons que l'intéraction répulsive entre les deux cations n'est pas évaluée par le modèle BVS dont l'une des limites est de tenir compte seulement de l'intéraction avec le réseau anionique. Ce concept ne tiend pas compte des intéractions cation-cation et anion-anion dans la structure cristalline comme dans le cas des travaux de (Ben Amor *et al.*, 2008).

*L*'étude structurale et l'analyse BVS permettent de conclure que la conduction dans ce matériau est probablement unidimensionnelle et dirigée selon l'axe b (Fig. 6).

L'examen des structures dans la série AMoO_2_AsO_4_ (A= cation monovalent) revèle que le cation monovalent a un rôle important dans l'arrangement structural. En effet, la substitution d'un cation monovalent par un autre plus petit peut conduire à différents systèmes cristallins centrosymétriques: (KMoO_2_AsO_4_, groupe d'espace *Pnma* (Zid & Jouini, 1996); β-NaMoO_2_AsO_4_, groupe *Pnma* (Ben Hlila *et al.*, 2009); RbMoO_2_AsO_4_, groupe Fddd (Zid & Jouini, 1999); AgMoO_2_AsO_4_, groupe *Pnma* (Hajji & Zid, 2006)). De plus, ce remplacement mène à des composés non-centrosymétriques (LiMoO_2_AsO_4_, groupe Pn2_1_a (Linnros, 1970); NaMoO_2_AsO_4_, groupe Pca2_1_ (Zid *et al.*, 1997); β-LiMoO_2_AsO_4_, groupe *P*2_1_ (Hajji *et al.*, 2004)). Ce balayage bibliographique montre que notre charpente est isostructurale aux matériaux KMoO_2_AsO_4_, AgMoO_2_AsO_4_ et β-NaMoO_2_AsO_4_. Elles diffèrent seulement par la disposition des cations monovalents dans la structure. Ce matériau mixte est construit aussi à partir de chaînes MoAsO_8_. Ce type de chaînes MXO_8_ est observé sous forme de VXO_8_ (*X*= P, As) dans d'autres composés rencontrés dans la bibliographie notamment: K_2_V_2_O_2_(AsO_4_)_2_ (Belkhiri *et al.*, 2012); KVPO_5_ (Benhamada *et al.*, 1991) et CaV_2_O(PO_4_)_2_ (Boudin *et al.*, 1995). Dans K_2_V_2_O_2_(AsO_4_)_2_ et KVPO_5_, les chaînes (VXO_8_)_∞_ se propagent selon les deux directions a et c dans lesquelles les tétraèdres AsO_4_ partagent leurs sommets avec quatre octaèdres VO_6_ et où les octaèdres VO_6_ se lient à quatre tétraèdres AsO_4_ et à deux octaèdres VO_6_ pour conduire à des chaînes infinies (VO_3_)_∞_. Cette association conduit à une structure tridimensionnelle. Contrairement à notre structure où les chaînes (MoAsO_8_)_∞_ se connectent par paires formant des chaînes doubles Mo_2_As_2_O_14_. D'autre part, dans CaV_2_O(PO_4_)_2_, les chaînes (VPO_8_)_∞_ se lient entre elles formant des doubles chaînes de type *M*_2_*X*_2_O_14_ (*M*=V, Mo et *X*=P, As) similaires à celles rencontrées dans K_0.78_Na_0.22_MoO_2_AsO_4_. Ces dernières se connectent à d'autres chaînes de type (VO_4_)_∞_ pour former des couches qui s'associent à leur tour pour conduire à une structure tridimensionnelle dans CaV_2_O(PO_4_)_2_.

### Experimental

Un mélange de K_2_CO_3_ (Fluka, 69858), Na_2_CO_3_ (Prolabo, 27778), (NH_4_)_2_Mo_4_O_13_ (Fluka, 69858) et NH_4_H_2_AsO_4_ (préparé au laboratoire, ASTM 01–775) pris dans les proportions K:Na:Mo:As=1:1:2:2 a été finement broyé dans un mortier en agate. Il a été mis dans un creuset et calciné à 573 K pendant 12 heures. Après refroidissement, le mélange a été de nouveau finement broyé puis porté à 803 K et maintenu à cette température pendant 4 jours. Le résidu final est refroidi lentement (5°K/demi journée, à 743 K) puis rapide (50°K/h) jusqu'à la température ambiante. Des cristaux jaunâtres sont apparus. Ils sont lavés à l'eau chaude afin de les séparer du flux. Les cristaux ont été ensuite triés sous une loupe binoculaire et l'échantillon sélectionné pour la collecte des données a été choisi après examen sous un microscope à lumière polarisée. *L*'analyse qualitative par la microscopie électronique à balayage de type FEI Quanta 200 d'un cristal sélectionné, confirme la présence des différents éléments chimiques attendus notamment: As, K, Mo, Na et l'oxygène.

### Refinement

*L*'utilisation de la fonction SUMP autorisée par le programme *SHELX*, pour les deux ions K1 et Na1 conduit à des ellipsoïdes bien définis. De plus, les densités d'électrons maximum et minimum restants dans la Fourier-différence sont acceptables et sont situées respectivements à 0,38 Å de O5 et à 1,05 Å de O4. S'il y a plus de deux composants d'un atome ou d'un groupe désordonné, il est nécessaire d'appliquer la fonction SUMP autorisée par le programme *SHELX*, utilisée pour représenter les taux d'occupation des variables libres. En effet, la fonction SUMP peut être appliquée aux valeurs d'entrée afin d'éviter les instabilités durant les étapes d'affinement. A cet effet, ces valeurs affinées sont affectées de numéros de paramètres immédiatement après les variables libres.

### Crystal data

**Table:**

K_0.78_Na_0.22_MoO_2_AsO_4_	*F*(000) = 561
*M**_r_* = 302.47	*D*_x_ = 4.117 Mg m^−^^3^
Orthorhombic, *P**n**m**a*	Mo *K*α radiation, λ = 0.71073 Å
Hall symbol: -P 2ac 2n	Cell parameters from 25 reflections
*a* = 10.5672 (9) Å	θ = 10–15°
*b* = 6.6323 (8) Å	µ = 10.05 mm^−^^1^
*c* = 6.9621 (8) Å	*T* = 298 K
*V* = 487.94 (9) Å^3^	Prism, yellow
*Z* = 4	0.45 × 0.33 × 0.23 mm

### Data collection

**Table:**

Enraf–Nonius CAD-4 diffractometer	553 reflections with *I* > 2σ(*I*)
Radiation source: fine-focus sealed tube	*R*_int_ = 0.037
Graphite monochromator	θ_max_ = 27.0°, θ_min_ = 3.5°
ω/2θ scans	*h* = −1→13
Absorption correction: ψ scan (North *et al.*, 1968)	*k* = −1→8
*T*_min_ = 0.025, *T*_max_ = 0.098	*l* = −8→8
1413 measured reflections	2 standard reflections every 120 min
576 independent reflections	intensity decay: 1.2%

### Refinement

**Table:**

Refinement on *F*^2^	Primary atom site location: structure-invariant direct methods
Least-squares matrix: full	Secondary atom site location: difference Fourier map
*R*[*F*^2^ > 2σ(*F*^2^)] = 0.025	*w* = 1/[σ^2^(*F*_o_^2^) + (0.0355*P*)^2^ + 0.6861*P*] where *P* = (*F*_o_^2^ + 2*F*_c_^2^)/3
*wR*(*F*^2^) = 0.067	(Δ/σ)_max_ < 0.001
*S* = 1.17	Δρ_max_ = 0.64 e Å^−^^3^
576 reflections	Δρ_min_ = −1.13 e Å^−^^3^
64 parameters	Extinction correction: *SHELXL*, Fc^*^=kFc[1+0.001xFc^2^λ^3^/sin(2θ)]^-1/4^
1 restraint	Extinction coefficient: 0.041 (2)

### Special details

**Table:**

Geometry. All e.s.d.'s (except the e.s.d. in the dihedral angle between two l.s. planes) are estimated using the full covariance matrix. The cell e.s.d.'s are taken into account individually in the estimation of e.s.d.'s in distances, angles and torsion angles; correlations between e.s.d.'s in cell parameters are only used when they are defined by crystal symmetry. An approximate (isotropic) treatment of cell e.s.d.'s is used for estimating e.s.d.'s involving l.s. planes.
Refinement. Refinement of *F*^2^ against ALL reflections. The weighted *R*-factor *wR* and goodness of fit *S* are based on *F*^2^, conventional *R*-factors *R* are based on *F*, with *F* set to zero for negative *F*^2^. The threshold expression of *F*^2^ > σ(*F*^2^) is used only for calculating *R*-factors(gt) *etc*. and is not relevant to the choice of reflections for refinement. *R*-factors based on *F*^2^ are statistically about twice as large as those based on *F*, and *R*- factors based on ALL data will be even larger.

### Fractional atomic coordinates and isotropic or equivalent isotropic displacement parameters (Å^2^)

**Table:**

	*x*	*y*	*z*	*U*_iso_*/*U*_eq_	Occ. (<1)
Mo1	0.33773 (3)	0.2500	0.04848 (5)	0.0073 (2)	
As1	0.15597 (4)	0.2500	0.46230 (6)	0.0063 (2)	
K1	0.0000	0.0000	1.0000	0.026 (2)	0.78 (4)
Na1	0.019 (4)	0.004 (7)	0.909 (13)	0.045 (8)	0.11 (2)
O1	0.4059 (3)	0.2500	0.8266 (5)	0.0162 (7)	
O2	0.5287 (3)	0.2500	0.1823 (5)	0.0112 (6)	
O3	0.1832 (3)	0.2500	0.9890 (5)	0.0221 (8)	
O4	0.1507 (2)	0.0449 (4)	0.6066 (3)	0.0135 (5)	
O5	0.2973 (3)	0.2500	0.3516 (4)	0.0108 (6)	

### Atomic displacement parameters (Å^2^)

**Table:**

	*U*^11^	*U*^22^	*U*^33^	*U*^12^	*U*^13^	*U*^23^
Mo1	0.0091 (3)	0.0092 (3)	0.0037 (3)	0.000	−0.00043 (12)	0.000
As1	0.0082 (3)	0.0069 (3)	0.0038 (3)	0.000	0.00037 (14)	0.000
K1	0.0228 (19)	0.0218 (13)	0.034 (5)	−0.0057 (8)	0.0014 (16)	−0.0102 (19)
Na1	0.050 (17)	0.039 (15)	0.05 (2)	−0.019 (11)	0.011 (16)	−0.025 (18)
O1	0.0176 (15)	0.0227 (17)	0.0082 (15)	0.000	0.0042 (12)	0.000
O2	0.0107 (12)	0.0181 (16)	0.0047 (14)	0.000	0.0007 (13)	0.000
O3	0.0126 (14)	0.042 (2)	0.0118 (16)	0.000	−0.0015 (14)	0.000
O4	0.0235 (13)	0.0091 (10)	0.0079 (10)	−0.0024 (8)	0.0005 (9)	0.0024 (8)
O5	0.0081 (13)	0.0167 (15)	0.0076 (14)	0.000	0.0014 (11)	0.000

### Geometric parameters (Å, º)

**Table:**

Mo1—O3^i^	1.685 (3)	K1—O2^viii^	2.781 (3)
Mo1—O1^i^	1.704 (3)	K1—O5^vii^	2.899 (2)
Mo1—O4^ii^	2.001 (2)	K1—O5^viii^	2.899 (2)
Mo1—O4^iii^	2.001 (2)	K1—O1^viii^	2.985 (3)
Mo1—O5	2.153 (3)	K1—O1^vii^	2.985 (3)
Mo1—O2	2.223 (3)	K1—O4^vi^	3.182 (2)
As1—O2^iv^	1.680 (3)	K1—O4	3.182 (2)
As1—O5	1.681 (3)	Na1—O2^viii^	2.36 (5)
As1—O4^v^	1.692 (2)	Na1—O3	2.45 (3)
As1—O4	1.692 (2)	Na1—O4	2.54 (9)
K1—O3^vi^	2.550 (3)	Na1—O5^viii^	2.60 (4)
K1—O3	2.550 (3)	Na1—O1^vii^	2.61 (7)
K1—O2^vii^	2.781 (3)	Na1—O3^vi^	2.81 (6)
			
O3^i^—Mo1—O1^i^	100.77 (16)	O5^viii^—K1—O1^viii^	73.00 (8)
O3^i^—Mo1—O4^ii^	96.26 (7)	O3^vi^—K1—O1^vii^	97.53 (8)
O1^i^—Mo1—O4^ii^	99.06 (7)	O3—K1—O1^vii^	82.47 (8)
O3^i^—Mo1—O4^iii^	96.26 (7)	O2^vii^—K1—O1^vii^	108.13 (6)
O1^i^—Mo1—O4^iii^	99.06 (7)	O2^viii^—K1—O1^vii^	71.87 (6)
O4^ii^—Mo1—O4^iii^	155.61 (13)	O5^vii^—K1—O1^vii^	73.00 (8)
O3^i^—Mo1—O5	92.79 (14)	O5^viii^—K1—O1^vii^	107.00 (8)
O1^i^—Mo1—O5	166.44 (13)	O3^vi^—K1—O4^vi^	62.19 (9)
O4^ii^—Mo1—O5	79.28 (7)	O3—K1—O4^vi^	117.81 (9)
O4^iii^—Mo1—O5	79.28 (7)	O2^vii^—K1—O4^vi^	54.95 (7)
O3^i^—Mo1—O2	169.45 (15)	O2^viii^—K1—O4^vi^	125.05 (7)
O1^i^—Mo1—O2	89.78 (14)	O5^vii^—K1—O4^vi^	51.47 (6)
O4^ii^—Mo1—O2	81.94 (6)	O5^viii^—K1—O4^vi^	128.53 (6)
O4^iii^—Mo1—O2	81.94 (6)	O1^viii^—K1—O4^vi^	57.25 (7)
O5—Mo1—O2	76.66 (12)	O1^vii^—K1—O4^vi^	122.75 (7)
O2^iv^—As1—O5	115.90 (16)	O3^vi^—K1—O4	117.81 (9)
O2^iv^—As1—O4^v^	109.23 (9)	O3—K1—O4	62.19 (9)
O5—As1—O4^v^	107.55 (9)	O2^vii^—K1—O4	125.05 (7)
O2^iv^—As1—O4	109.23 (10)	O2^viii^—K1—O4	54.95 (7)
O5—As1—O4	107.55 (9)	O5^vii^—K1—O4	128.53 (6)
O4^v^—As1—O4	107.03 (16)	O5^viii^—K1—O4	51.47 (6)
O3^vi^—K1—O2^vii^	116.53 (10)	O1^viii^—K1—O4	122.75 (7)
O3—K1—O2^vii^	63.47 (10)	O1^vii^—K1—O4	57.25 (7)
O3^vi^—K1—O2^viii^	63.47 (10)	O2^viii^—Na1—O3	141 (4)
O3—K1—O2^viii^	116.53 (10)	O2^viii^—Na1—O4	69 (2)
O3^vi^—K1—O5^vii^	78.48 (7)	O3—Na1—O4	74.2 (19)
O3—K1—O5^vii^	101.52 (7)	O2^viii^—Na1—O5^viii^	66.1 (13)
O2^vii^—K1—O5^vii^	57.05 (9)	O3—Na1—O5^viii^	86.5 (11)
O2^viii^—K1—O5^vii^	122.95 (9)	O4—Na1—O5^viii^	62.1 (16)
O3^vi^—K1—O5^viii^	101.52 (7)	O2^viii^—Na1—O1^vii^	86 (2)
O3—K1—O5^viii^	78.48 (7)	O3—Na1—O1^vii^	93 (2)
O2^vii^—K1—O5^viii^	122.95 (9)	O4—Na1—O1^vii^	70 (2)
O2^viii^—K1—O5^viii^	57.05 (9)	O5^viii^—Na1—O1^vii^	131 (4)
O3^vi^—K1—O1^viii^	82.47 (8)	O2^viii^—Na1—O3^vi^	65.1 (9)
O3—K1—O1^viii^	97.53 (8)	O3—Na1—O3^vi^	152 (4)
O2^vii^—K1—O1^viii^	71.87 (6)	O4—Na1—O3^vi^	134 (2)
O2^viii^—K1—O1^viii^	108.13 (6)	O5^viii^—Na1—O3^vi^	102.7 (16)
O5^vii^—K1—O1^viii^	107.00 (8)	O1^vii^—Na1—O3^vi^	100.6 (10)

Symmetry codes: (i) *x*, *y*, *z*−1; (ii) −*x*+1/2, *y*+1/2, *z*−1/2; (iii) −*x*+1/2, −*y*, *z*−1/2; (iv) *x*−1/2, *y*, −*z*+1/2; (v) *x*, −*y*+1/2, *z*; (vi) −*x*, −*y*, −*z*+2; (vii) *x*−1/2, *y*, −*z*+3/2; (viii) −*x*+1/2, −*y*, *z*+1/2.
